# Modeling the Impact of Increased Adherence to Asthma Therapy

**DOI:** 10.1371/journal.pone.0051139

**Published:** 2012-12-07

**Authors:** Amory Schlender, Peter E. Alperin, Helene L. Grossman, E. Rand Sutherland

**Affiliations:** 1 Archimedes Incorporated, San Francisco, California, United States of America; 2 Department of Medicine, National Jewish Health, Denver, Colorado, United States of America; 3 Department of Medicine, University of Colorado, Denver, Colorado, United States of America; Leiden University Medical Center, The Netherlands

## Abstract

**Background:**

Nonadherence to medications occurs in up to 70% of patients with asthma. The effect of improving adherence is not well quantified. We developed a mathematical model with which to assess the population-level effects of improving medication prescribing and adherence for asthma.

**Methods:**

A mathematical model, calibrated to clinical trial data from the U.S. NHLBI-funded SOCS trial and validated using data from the NHLBI SLIC trial, was used to model the effects of increased prescribing and adherence to asthma controllers. The simulated population consisted of 4,930 individuals with asthma, derived from a sample the National Asthma Survey. Main outcomes were controller use, reliever use, unscheduled doctor visits, emergency department (ED) visits, and hospitalizations.

**Results:**

For the calibration, simulated outcomes agreed closely with SOCS trial outcomes, with treatment failure hazard ratios [95% confidence interval] of 0.92 [0.58–1.26], 0.97 [0.49–1.45], and 1.01 [0–1.87] for simulation vs. trial in the in placebo, salmeterol, and triamcinolone arms, respectively. For validation, simulated outcomes predicted mid- and end-point treatment failure rates, hazard ratios 1.21 [0.08–2.34] and 0.83 [0.60–1.07], respectively, for patients treated with salmeterol/triamcinolone during the first half of the SLIC study and salmeterol monotherapy during the second half. The model performed less well for patients treated with salmeterol/triamcinolone during the entire study duration, with mid- and end-point hazard ratios 0.83 [0.00–2.12] and 0.37 [0.10–0.65], respectively. Simulation of optimal adherence and prescribing indicated that closing adherence and prescription gaps could prevent as many as nine million unscheduled doctor visits, four million emergency department visits, and one million asthma-related hospitalizations each year in the U.S.

**Conclusions:**

Improvements in medication adherence and prescribing could have a substantial impact on asthma morbidity and healthcare utilization.

## Introduction

Asthma is a prevalent chronic disease which impacts 7.7% of US adults and 9.4% of US children [1], accounting for 17 million ambulatory care visits [2] and 456,000 hospitalizations per year [3]. According to national and international guidelines, one main goal of asthma therapy is to control airway inflammation, and thereby increase control of symptoms [4]. A number of effective therapies for managing airway inflammation are available, both inhaled corticosteroids (ICS) and long-acting beta-2 agonists have been shown to improve lung function, decrease rescue bronchodilator use, increase asthma-specific quality of life, and decrease asthma-related healthcare utilization.

Despite their therapeutic efficacy, the use of controller medications falls short of the levels recommended by guidelines. Studies examining corticosteroid adherence amongst patients with difficult-to-treat asthma in clinical settings have shown nonadherence, either partial or full, in up to 70% of patients with an active controller prescription [5], [6]. Studies of the impact of nonadherence on outcomes have been made difficult by the relatively low frequency of asthma exacerbations and related events such as oral CS use, emergency department (ED) visits or hospitalizations. Additionally, the episodic nature of asthma can lead to intermittent ICS use, with increases in use in the context of worsening control potentially even suggesting reverse causation.

Data from the 2005 National Asthma Survey (NAS), including medication use, symptoms, and healthcare utilization for patients with physician-diagnosed asthma, show that 27% of patients whom guidelines would deem eligible for a controller medication do not have an active prescription. Medication nonadherence occurs in patients with a wide range of disease severities [5], and interventions which increase patient adherence to therapy have been shown to be beneficial with regard to important clinical parameters such as lung function, rescue bronchodilator use, asthma-specific quality of life, and asthma-related healthcare utilization [7]. In one of the few papers to quantitate the impact of nonadherence in asthma, Williams and colleagues estimated that every 25% increase in adherence was associated with a 11% reduction in the risk of asthma exacerbation. Furthermore, the authors estimated that almost 25% of severe asthma exacerbations could be prevented with improved ICS adherence [8].

For healthcare decision makers, it would be valuable to quantify the effects of improved adherence to, and expanded prescription of, controller therapies for asthma, especially in the general asthmatic population. While the strongest form of evidence would come from a clinical trial, no existing trials address these questions directly. In cases such as this one, mathematical models can provide insight by exploring scenarios where actual trials are unfeasible because of limitations in time or resources, ethical constraints, or other factors.

We have constructed a model of asthma that is designed to simulate the effects of therapeutic interventions on subsets of the general asthmatic population. This model is one of several components of the Archimedes Model of healthcare, a computational model designed to help decision makers examine the implications of population-scale interventions in chronic diseases, including cancer, coronary artery disease, hypertension, and diabetes [9]–[11]. In this report, we describe how we constructed, calibrated, and validated the asthma model. We then demonstrate a use of this model by exploring the implications of expanded prescription of, and improved adherence to, controller medications amongst a representative subset of the US population with asthma.

## Methods

The Archimedes Model is a stochastic simulation model which incorporates substantial anatomic, physiologic, clinical, and administrative detail. The mathematical formulation of the Model has been described previously [11]. We developed a stand-alone asthma model, which borrows methods from the Archimedes model but focuses on the specific clinical and physiologic aspects of asthma.

### Incorporation of Asthma Variability into the Model

**Figure 1:**
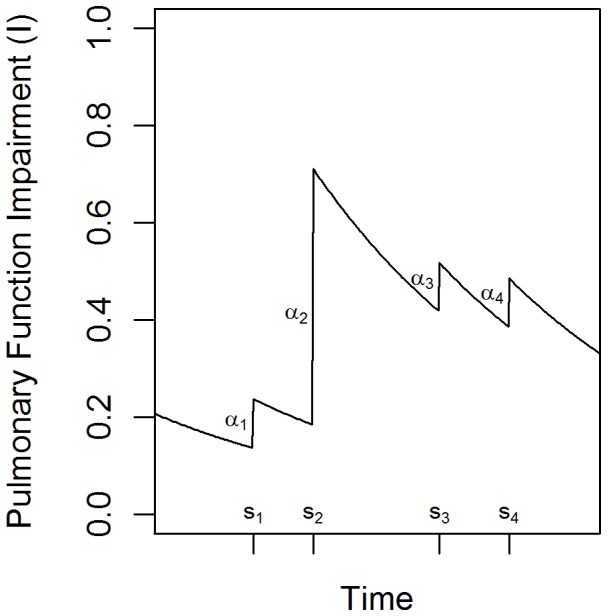
Schematic of modeled physiological progression through time. An example of physiological progression through time, evolving according to Equation 1, with stimulus event times and effect sizes labeled as s_i_ and *α_i_*, respectively.

Because of the variable nature of airflow limitation and symptoms in asthma, day-to-day fluctuations in environmental exposures and airflow limitation were modeled (Figure 1). *In silico*, individually modeled members of the study population are exposed to environmental triggers (stimuli) over time, resulting in an increase in magnitude of their degree of pulmonary function impairment. Between stimulus events, each individual’s physiology returns toward a normal, unimpaired state. The core equation of the model describes this process as:


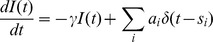
, where *t* is time, *I* is a function describing pulmonary function impairment due to inflammation, *γ* is the rate of return, as observed in the FACET trial [12], toward a normal, unimpaired state, *δ* is a Dirac delta function, and *α_i_* and *s_i_* are the effect size and time, respectively, of the *i^th^* trigger event. Stimulus events are generated stochastically using a Poisson process, with the effect size of each event depending on the affected individual’s sensitivity to that stimulus.

### Incorporation of Treatment Interventions into the Model

**Figure 2:**
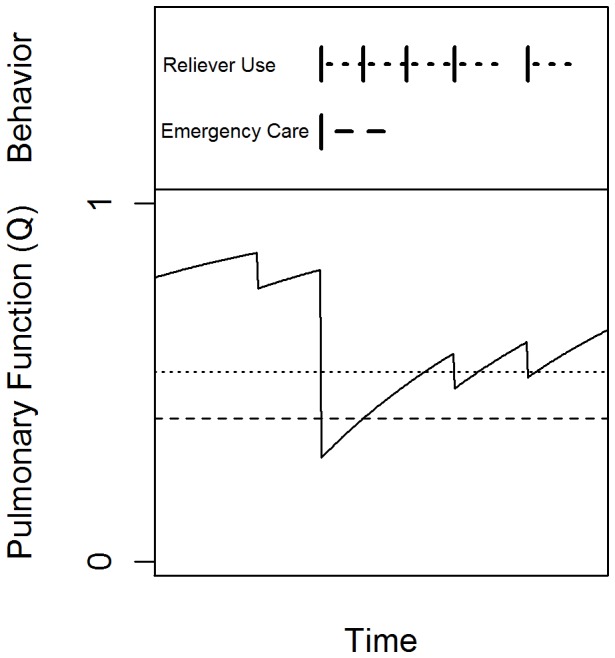
Schematic of patient behavior as a function of pulmonary function and behavioral thresholds. The patient initiates relief medication when his or her pulmonary function (solid line) falls below his or her relief medication threshold (dotted line), and seeks emergency care when his or her pulmonary function falls below his or her emergency care threshold (dashed line).

Clinically, an increase in the degree of airflow limitation is often accompanied by an increase in symptoms of asthma, and patients are counseled to respond to these reductions in pulmonary function by using additional reliever and/or controller medications and seeking medical care. To capture this behavior, we further constructed the model to reflect the initiation of reliever (inhaled short-acting β_2_-agonists) and controller (inhaled or systemic glucocorticoids, inhaled long-acting β_2_-agonists) therapy and the use of unscheduled medical care once a modeled individual’s lung function dropped below a particular threshold (Figure 2). In modeled individuals, pulmonary function *Q* depends on *I* and a bronchodilator factor, σ, as: 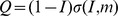, where *Q = 1* corresponds to the upper limit of the individual’s pulmonary function, *Q = 0* corresponds to the lower limit of achievable pulmonary function, and σ depends on airway inflammation, *I*, and an active treatment intervention, *m*. Absent medication, *σ* = 1, corresponding to no bronchodilator effect.

The model incorporates the effect of guideline-recommended medications for the control and relief of asthma symptoms, including short-acting β_2_-agonists (SABA), inhaled corticosteroids (ICS), long-acting β_2_-agonists (LABA), and oral corticosteroids (OCS). β_2_-agonist medications affect the bronchodilator factor *σ*, improving pulmonary function *Q*. Corticosteroids are modeled with an anti-inflammatory effect, modifying *γ* and the sensitivity parameters governing *α_i_* in Equation 1, thereby accelerating patients’ rate of return toward a normal state and reducing sensitivity to stimuli. Both classes of medications are modeled to take effect instantaneously, with a constant effect over their reported duration of action. However, because corticosteroid medications only affect the rate of change of *I*, only β_2_-agonist medications have an immediate effect on patients’ level of pulmonary function.

The use of unscheduled emergency care is modeled phenomenologically, with patients’ pulmonary function held stable during inpatient encounters. Fixed probabilities and duration of admission to hospital [13], [14] irrespective of pulmonary function at presentation [15], are incorporated into the model, and after discharge all modeled individuals receive a course of oral steroids.

### Training, Calibration, and Validation of the Model

To demonstrate that the model can be calibrated to capture the effects of standard asthma medications, we simulated the population and treatment protocols of the SOCS trial [16] using data obtained from the NHLBI BioLINCC website [17]. The simulated population incorporated clinical data from the SOCS study population including history of controller medication use and unscheduled care encounters in previous year, and pulmonary function measures and rescue medication use patterns observed during the trial’s run-in period. The simulation’s target population was derived from individual-level data from the SOCS trial, randomly sampled with replacement to include 300 individuals drawn from the 164 SOCS participants. While each virtual patient’s physiological and behavioral features were calibrated with respect to this target population, the stochastic nature of the calibration process meant that if the same SOCS individual were calibrated multiple times, each copy of the person would have a different parameterization. Moreover, because of the simulation’s stochastic nature, a calibrated person simulated multiple times would not be expected to experience the same series of outcomes during each simulation. Therefore, while the model captured the overall distribution of risk in the SOCS population, simulated individuals would not be expected to reproduce the specific outcomes of each individual in the SOCS trial. Accordingly, we assessed the correspondence between the simulated and real patients at a population, rather than an individual, level. The run-in and active treatment periods of the SOCS trial, as previously described, were then simulated and the results were aggregated by treatment arm. Treatment parameters were tuned iteratively over a succession of SOCS simulations with a goal of matching the treatment failure rates observed in each of the original SOCS trial’s treatment arms. In these simulations, treatment failure was defined as a patient's pulmonary function dropping below the threshold at which he or she would normally seek immediate care from his or her personal physician. Rescue inhaler-use rates were included as a secondary calibration target.

To validate the model’s predictive utility, we simulated the population and treatment protocols of the SLIC trial [18] using the ICS and LABA models developed by calibration to SOCS, again with data obtained from the NHLBI BioLINCC website. This simulation’s target population was derived from individual-level data from the SLIC trial, randomly sampled with replacement to include 300 individuals drawn from the 175 SLIC participants. As with our simulated SOCS population, the parameterization of each simulated SLIC patient was unique. The run-in and active treatment periods of the SLIC trial, as previously described, were then simulated and results were aggregated by treatment arm. Simulation results were compared to the treatment failure rate and rescue inhaler use rate observed in SLIC.

To assess its utility as a forecasting tool, we used the model to investigate three idealized treatment scenarios in a general, nationally representative population of asthma patients based on the National Asthma Survey (NAS) [13]. Our target population was based on a weighted sample of 4,930 NAS patients with an active history of controller and/or relief medication use, as described in Table 1. For each patient in the target population, rates of unscheduled doctor and ED visits, hospital stays, controller medication use, and rescue inhaler use were taken directly from NAS. Pulmonary function measures and rescue inhaler use patterns were imputed from the SLIC and SOCS datasets. For patients taking a controller medication, adherence was estimated by comparing self-reported use rates to daily or twice-daily use, as recommended by guidelines. As a sensitivity analysis for our ICS model, the population was simulated three times, with three different ICS effect sizes. Baseline ICS effect was derived from the SLIC trial, with the effect of ICS on pulmonary function increased and decreased, respectively, by 20% for a pair of sensitivity analyses. In all three effect size cases, a unique virtual population was calibrated as described previously.

**Table 1:** General characteristics of our simulated asthmatic population.

Characteristics	All	Age 18+	Age 12–17
Simulated Population Size	4,930	4,090	837
Age, mean (SD)	38.5 (18.32)	43.4 (16.2)	14.6 (1.6)
Male, %	61.8	63.8	52.1
FEV1 (L) mean (SD)	2.71 (0.71)	2.70 (0.71)	2.75 (0.72)
FEV1% predicted, mean (SD)	80.4 (14.5)	80.4 (14.5)	80.5 (14.8)
Active Smoker, %*	–	20.25	–
ICS Use, % [% Adherence^†^]	43.2 [80.4]	42.5 [81.5]	46.4 [75.7]
Both ICS and LABA Use, %[% Adherence^†^]	22.6 [85.1]	22.0 [84.2]	25.1 [88.9]
Qualifies for additional ICSprescription, %	27.1	28.5	20.6
Rescue Albuterol Uses/Day,mean (SD)	1.11 (1.42)	1.23 (1.47)	0.57 (0.92)
Acute Care Encounters,mean (SD):			
Unscheduled Dr. Visits inPast Year	1.49 (2.94)	1.56 (2.94)	1.12 (2.89)
ER/UC Visits in Past Year	0.56 (1.64)	0.57 (1.69)	0.51 (1.63)
Hospital Stays in Past Year	0.15 (0.71)	0.17 (0.75)	0.06 (0.50)

The population was derived by sampling from the National Asthma Survey (NAS), with FEV_1_ (absolute and % predicted) imputed using individual spirometry data taken from the run-in period of the SLIC and SOCS trials. All other values are taken directly from NAS.

Adherence (^†^) is defined as the fraction of days of use for patients using a particular medication.

Smoking status (*) modeled in adults only, based on NAS data.

In order to compare the relative public health impact of increasing the number of prescriptions of controller medications across the population with improving adherence to prescriptions already written, we simulated four different treatment groups: currently observed prescribing and adherence (based on NAS) (OO), currently observed prescribing but perfect adherence (OP), expanded guideline-based prescribing with currently observed adherence (EO), and expanded prescribing with perfect adherence (EP). In the OO scenario, patients are prescribed and adhere to controller medications as observed in their NAS counterparts. In the OP scenario, all patients with an ICS prescription use their controller twice daily, as recommended by guidelines. In the EO scenario, all patients whose NAS counterpart did not report ICS use but who experienced one or more serious exacerbations in the past year or used their rescue inhaler twice or more per day, on average, are prescribed a medium-dose ICS, and adhere to this regimen at the rate observed amongst current ICS users in NAS. The EP population consisted of perfectly adherent EO patients. All patients were simulated four times, once under each treatment scenario, for one year.

## Results

### Fidelity of the Model in Predicting Outcomes in SOCS and SLIC

**Figure 3:**
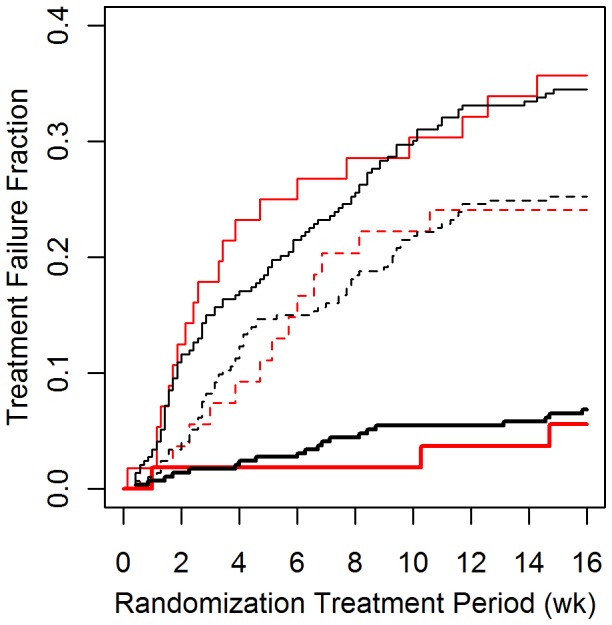
Treatment failure rates in the SOCS trial. Treatment failure vs. time for the SOCS trial (red), and our simulation thereof (black), shown by treatment arm. Rates are highest in the placebo arm (thin lines), followed by the salmeterol arm (dashed lines), and the triamcinolone arm (thick lines). The difference between simulated and actual failure rates was not statistically significant (log-rank P = 0.22, 0.40, and 0.95 for difference in failure rates between simulation and trial under the placebo, salmeterol, and triamcinolone treatment conditions, respectively).

The rate of model-simulated treatment failures agreed closely with treatment failure rates observed in the SOCS trial, as shown in Figure 3. There were no significant differences between the rates observed in our simulation and the actual trial, with end-point hazard ratios [95% confidence interval] of 0.92 [0.58–1.26], 0.97 [0.49–1.45], and 1.01 [0–1.87] between simulated and trial failure rates for the placebo, salmeterol, and ICS treatment arms, respectively.

**Figure 4:**
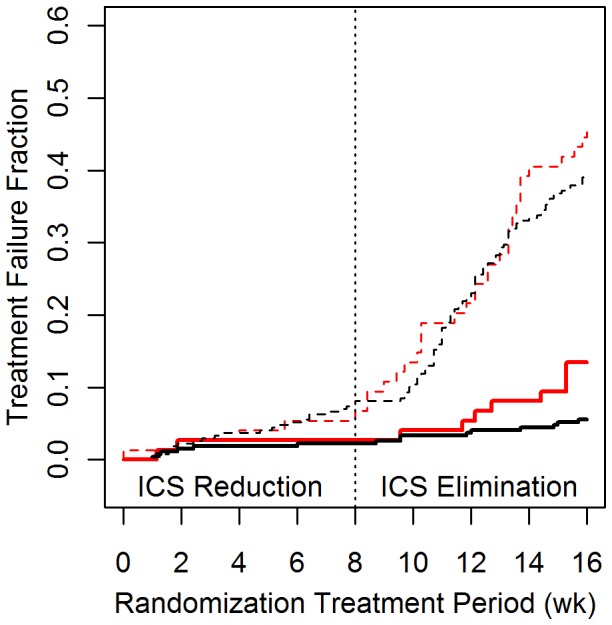
Treatment failure rates in the SLIC trial. Treatment failure vs. time for the SLIC trial (red), and our simulation thereof (black), shown by treatment arm. Treatment failures were more frequent in the salmeterol monotherapy arms (dashed lines) and less frequent in the salmeterol/triamcinolone arms (solid lines). During the ICS reduction phase, simulated failure rates were not significantly different from those observed in the trial in either the salmeterol/triamcinolone (P = 0.71) or salmeterol monotherapy (P = 0.56) arms. During the ICS elimination phase, the simulation underpredicted the trial’s treatment failure rate in the salmeterol/triamcinolone arm (P = 0.04), but showed no significant difference from observed in the salmeterol monotherapy arm (P = 0.76).

The rate of model-simulated treatment failures agreed closely with treatment failures observed in the SLIC salmeterol minus arm, but did not match the end-point failure rate in the salmeterol plus trial arm, as shown in Figure 4. In the salmeterol minus arm, where steroid treatment was terminated during the second-half of the study, treatment failure hazard ratios were 1.21 [0.08–2.34] and 0.83 [0.60–1.07] at the trial’s mid- and end-points, respectively. In the salmeterol plus arm, where steroid treatment continued throughout the study, treatment failure hazard ratios were 0.83 [0.00–2.13] and 0.37 [0.10–0.65] at the trial’s mid- and end-points, respectively.

### Modeling the Effects of Increases in Controller Prescribing and Adherence

Baseline characteristics for the 4,930 simulated patients derived from the NAS sample are shown in Table 1. The modeled population is predominantly adult, with lung function impairment reflected by FEV_1_ of 80.4 (14.5) % predicted, regular use of albuterol at 1.11 (1.4) puffs/day, and an average of 1.49 (2.94) unscheduled physician visits/year. Results of a one-year simulation of the population under each combination of prescription and adherence scenarios are shown in Table 2. As compared with all other scenarios, rates of reliever use, unscheduled doctor visits, ED visits, and hospitalizations were highest under the currently observed prescription/adherence (OO) scenario, and lowest under the expanded prescription/perfect adherence (EP) scenario (P<0.02). Simulating perfect adherence in the 43% of participants prescribed a controller (OP) reduced the need for rescue bronchodilators by 22.6%, reduced unscheduled physician visits by 27.4%, and reduced ED visits and hospitalizations by 46.3 and 47.1%, respectively. The effect of increasing controller prescriptions to guideline-recommended levels, while holding adherence rates stable (the EO scenario), resulted in effects nearly identical to those observed in the OP scenario (p range from comparisons between 0.2 and 1.0), except for the rate of ED utilization, which was lower in the OP scenario than in the EO scenario (P = 0.02). The greatest effects were observed with increasing both prescribing (to guideline levels) and adherence (to 100%). In this scenario (EP), reliever use was reduced by 53.2%, unscheduled doctor visits were reduced by 64.4%, and ED visits and hospitalizations were reduced by 72.2 and 76.5%, respectively. Results were not sensitive to ICS effect size.

**Table 2:** Average simulated outcome rates, given differential prescribing and adherence scenarios, for asthmatic patients using inhaled controller or relief medications in the US.

	ObservedPrescribingObservedAdherence (OO)	ObservedPrescribingPerfectAdherence (OP)	ExpandedPrescribingObservedAdherence (EO)	ExpandedPrescribingPerfectAdherence (EP)
Controller users, %	43.2	43.2	70.4	70.4
Mean controller uses per day (SD)	0.27 (0.02)	0.43 (0.03)	0.40 (0.02)	0.70 (0.02)
Mean reliever uses, per day (SD)	1.41 (0.07)	1.09 (0.06)	1.14 (0.07)	0.66 (0.04)
Mean urgent care visits, per year (SD)	1.46 (0.11)	1.06 (0.08)	1.06 (0.1)	0.52 (0.05)
Mean ER visits, per year (SD)	0.54 (0.08)	0.29 (0.04)	0.43 (0.08)	0.15 (0.03)
Mean hospitalizations, per year (SD)	0.17 (0.03)	0.09 (0.01)	0.13 (0.02)	0.04 (0.01)

Major respiratory outcome rates for patients simulated under each of four prescription/adherence scenarios. All differences in outcome rates observed under different scenarios were significant (p<0.03), with the exception of controller use, reliever use, urgent office visits, and hospitalizations in the OP and EO scenarios. Because each treatment scenario is simulated with the same pool of patients, individual-level outcomes under each treatment condition are highly correlated.

Table 3 shows the estimated impact of increased corticosteroid use under the EO, OP, and EP scenarios in averting, both in absolute number and percent reduction, unscheduled doctor visits, ED visits, and hospitalizations. Although the EO and OP scenarios had a similar effect with regard to reduction in unscheduled visits, at the population level the OP scenario resulted in greater use of ICS and greater reduction in ER visits and hospitalizations. The greatest degree of benefit was seen with the EP scenario, which yielded a 60% reduction in unscheduled visits, a 70% reduction in ER visits, and an 80% reduction in overnight hospitalizations.

**Table 3:** Predicted number of asthma exacerbation events.

Scenario	Number of PatientsReceiving Additional ICS	Unscheduled Visits Per Year	ED Visits Per Year	Hospitalizations Per Year
		*Current NAS self-reported event rate*		
OO	0	14,000,000	5,200,000	1,400,000
		*Predicted number (%) of events averted relative to NAS rate*		
EO	2,500,000	3,700,000 (30%)	1,000,000 (20%)	300,000 (20%)
OP	4,000,000	3,800,000 (30%)	2,500,000 (50%)	700,000 (50%)
EP	6,500,000	8,800,000 (60%)	3,500,000 (70%)	1,100,000 (80%)

Predicted number of exacerbation-related utilization events under the current standard of care (OO), and number (%) of events averted relative to OO under each of our idealized treatment scenarios.

## Discussion

The applicability of clinical trial data to real-world populations has long been debated, and in the case of asthma, the selection criteria of typical randomized controlled trials have been reported to exclude upwards of 90% of asthmatic patients [19]. This has led to the recent emergence of pragmatic trials in asthma [20], which use observational approaches to test efficacy in larger heterogeneous populations and may provide data relevant to a larger proportion of real-world patients [21]. In this report, we propose an alternative approach to evaluating population-level interventions in asthma which uses a model initially based on clinical trial data and then applied to modeled real-world populations derived from survey data, which rely largely on data self-reported by patients.

Using such an approach based on the validated Archimedes Model, we were successful in calibrating the asthma model’s prediction of ICS and LABA effects to match the treatment failure rates observed in the SOCS trial and found that, using these treatment effect models, the asthma model successfully predicted the outcomes reported in the SLIC trial. Having developed a model that accurately predicted the clinical effects of ICS and LABA, two widely used controller medications, we then applied that model to a population derived from the National Asthma Survey to quantify the effects of increasing prescriptions to guideline-recommended levels, increasing adherence, or both, on the frequency of important markers of asthma control and healthcare utilization, including rescue bronchodilator use, unscheduled care visits, ED visits, and hospitalizations.

As reported, we observed that improvements in medication adherence and prescription could lead to significant improvements in asthma outcomes. Interestingly, the overall increase in the rates of controller use were comparable under the OP and EO scenarios, giving us the opportunity to assess the relative effectiveness of these different strategies for increasing controller use rates. While the two strategies were comparable with regard to reductions in rates of reliever use and unscheduled doctor visits, on a per-use basis it appears that improving patient adherence to controller therapy is substantially more effective at preventing ED visits and hospital stays than expanding prescriptions. This may indicate that clinicians have already successfully provided an asthma controller to many of the patients at highest risk for serious asthma exacerbations, but that further improvements in adherence could bring about significant improvements in control and lead to significant savings in both cost and healthcare utilization in the population in general. Additional gains could be expected if both prescribing and adherence were optimized. However, the feasibility of substantially improving adherence must be considered; while the expanded prescription/observed adherence scenario shows the smallest overall benefit of the three strategies, it is perhaps the most feasible, as it targets the fewest people and assumes no changes in adherence behaviors, rather relying on changing the prescription norms among physicians.

These data are the result of a model, rather than an observational or controlled study; thus, there are limitations. To improve confidence in the model for populations of patients with asthma, the model’s predictions should be compared to a broader portfolio of clinical studies. While there have been advances in making the data from clinical trials publicly available, limited access to the sort of individual-level clinical trial data used herein is a significant obstacle. Along these lines, the simulation is also subject to the limits of our modeling assumptions and training data. Any biases in the NAS, SOCS, or SLIC data, will be expressed by the model. Further, any feature (such as pulmonary function) that is not included in major asthma surveillance datasets can be included only by imputation across datasets, as performed here, and can lead to substantial uncertainty in results. Thus, additional validation efforts are needed.

In conclusion, mathematical models can play the important role of integrating evidence collected during clinical trials and other primary investigations, and extending these specific observations to explore their implications in a broad range of patient populations. We have demonstrated that the Archimedes asthma model can be calibrated to capture the outcome rates observed in clinical trials, and that these results can be extended to investigate asthma morbidity in general populations. Our simulation predicts that closing adherence and prescription gaps among asthma patients in the United States could prevent as many as nine million unscheduled doctor visits, four million ED visits, and one million asthma-related hospitalizations each year. Future applications include the cost effectiveness of different treatment strategies, assessing the comparative effectiveness of novel therapies and the implications of changes in treatment guidelines. Thus, mathematical models combining aspects of clinical trial and real-world data can help provide insight into the comparative effectiveness of novel therapies and the public health implications of changes in treatment guidelines.
